# High prevalence of cardiometabolic risk factors amongst young adults in the United Arab Emirates: the UAE Healthy Future Study

**DOI:** 10.1186/s12872-023-03165-3

**Published:** 2023-03-15

**Authors:** Fatima Mezhal, Abderrahim Oulhaj, Abdishakur Abdulle, Abdulla AlJunaibi, Abdulla Alnaeemi, Amar Ahmad, Andrea Leinberger-Jabari, Ayesha S. Al Dhaheri, Eiman AlZaabi, Fatma Al-Maskari, Fatme Alanouti, Fayza Alameri, Habiba Alsafar, Hamad Alblooshi, Juma Alkaabi, Laila Abdel Wareth, Mai Aljaber, Marina Kazim, Michael Weitzman, Mohammad Al-Houqani, Mohammad Hag Ali, E. Murat Tuzcu, Naima Oumeziane, Omar El-Shahawy, Rami H. Al-Rifai, Scott Sherman, Syed M. Shah, Thekra Alzaabi, Tom Loney, Wael Almahmeed, Youssef Idaghdour, Luai A. Ahmed, Raghib Ali

**Affiliations:** 1 Public Health Research Center, New York University Abu Dhabi, Abu Dhabi, UAE; 2 Department of Epidemiology and Public Health, College of Medicine and Health Sciences, Khalifa University of Sciences and Technology, Abu Dhabi, UAE; 3 Department of Pediatrics, Zayed Military Hospital, Abu Dhabi, UAE; 4 Department of Cardiology, Zayed Military Hospital, Abu Dhabi, UAE; 5 Department of Nutrition and Health, College of Medicine and Health Sciences, UAE University, Al-Ain, UAE; 6 Department of Pathology, Sheikh Shakhbout Medical City, Abu Dhabi, UAE; 7 Institute of Public Health, College of Medicine and Health Sciences, UAE University, Al-Ain, UAE; 8 Zayed Center for Health Sciences, UAE University, Al-Ain, UAE; 9 College of Natural and Health Sciences, Zayed University, Abu Dhabi, UAE; 10 Zayed Military Hospital, Abu Dhabi, UAE; 11 Center for Biotechnology, Khalifa University of Science and Technology, Abu Dhabi, UAE; 12 Department of Genetics and Molecular Biology, Khalifa University of Science and Technology, Abu Dhabi, United Arab Emirates; 13 Department of Biomedical Engineering, Khalifa University of Science and Technology, Abu Dhabi, United Arab Emirates; 14 Abu Dhabi Blood Bank Services, SEHA, Abu Dhabi & Al-Ain,, UAE; 15 Department of Internal Medicine, College of Medicine and Health Sciences, UAE University, Al-Ain, UAE; 16 Pathology and Laboratory Medicine Institute, Cleveland Clinic Abu Dhabi, Abu Dhabi, UAE; 17 Healthpoint Hospital, Abu Dhabi, UAE; 18 Department of Environmental Medicine, New York University of Medicine, New York, USA; 19 Department of Medicine, College of Medicine and Health Sciences, UAE University, Al-Ain, UAE; 20 Department of Health Science, Higher Colleges of Technology, Abu Dhabi, UAE; 21 Heart and Vascular Institute, Cleveland Clinic Abu Dhabi, Abu Dhabi, UAE; 22 Department of Population Health, New York University School of Medicine, New York, USA; 23 College of Medicine, Mohammed Bin Rashid University of Medicine and Health Sciences, Dubai, UAE; 24 MRC Epidemiology Unit, University of Cambridge, Cambridge, UK

**Keywords:** Non-communicable disease, Cardiovascular disease, Cardiometabolic risk factors, Obesity, Dysglycemia, Dyslipidemia, Hypertension, Central obesity

## Abstract

**Background:**

Cardiovascular disease (CVD) is the leading cause of death in the world. In the United Arab Emirates (UAE), it accounts for 40% of mortality. CVD is caused by multiple cardiometabolic risk factors (CRFs) including obesity, dysglycemia, dyslipidemia, hypertension and central obesity. However, there are limited studies focusing on the CVD risk burden among young Emirati adults. This study investigates the burden of CRFs in a sample of young Emiratis, and estimates the distribution in relation to sociodemographic and behavioral determinants.

**Methods:**

Data was used from the baseline data of the UAE Healthy Future Study volunteers. The study participants were aged 18 to 40 years. The study analysis was based on self-reported questionnaires, anthropometric and blood pressure measurements, as well as blood analysis.

**Results:**

A total of 5167 participants were included in the analysis; 62% were males and the mean age of the sample was 25.7 years. The age-adjusted prevalence was 26.5% for obesity, 11.7% for dysglycemia, 62.7% for dyslipidemia, 22.4% for hypertension and 22.5% for central obesity. The CRFs were distributed differently when compared within social and behavioral groups. For example, obesity, dyslipidemia and central obesity in men were found higher among smokers than non-smokers (*p* < 0.05). And among women with lower education, all CRFs were reported significantly higher than those with higher education, except for hypertension. Most CRFs were significantly higher among men and women with positive family history of common non-communicable diseases.

**Conclusions:**

CRFs are highly prevalent in the young Emirati adults of the UAE Healthy Future Study. The difference in CRF distribution among social and behavioral groups can be taken into account to target group-specific prevention measures.

**Supplementary Information:**

The online version contains supplementary material available at 10.1186/s12872-023-03165-3.

## Background

Cardiovascular diseases (CVD) are the most common non-communicable diseases (NCDs) globally, and constitute the leading cause of global mortality, as well as a major contributor to reduced quality of life [1]. CVD death rates have increased steadily from 12.1 million, in 1990, to 18.6 million in 2019 [2]. In 2017, it was responsible for 17.8 million deaths worldwide and corresponded to 35.6 million years lived with related disability [1, 2]. Approximately 80% of CVD-related deaths are caused by coronary heart disease (CHD), and strokes. The World Health Organization (WHO) reports that NCDs account for 77% of all deaths in the United Arab Emirates (UAE); CVDs account for 40% of the causes [3]. The UAE Ministry of Health and Prevention report (2019) revealed that 22% of CVD-related deaths were attributable to acute myocardial infarction, followed by cerebrovascular disease, ischemic heart disease, and hypertension [4].

Individuals at risk of CVD may have a cluster of risk factors including obesity, raised blood pressure, high blood glucose, abnormal lipids as well as abdominal obesity. These are the most common cardiovascular risk factors, also referred to as cardiometabolic risk factors (CRFs). The INTERHEART study, which included data from 52 countries across the world, showed that smoking, hypertension, high low-density lipoprotein level, and diabetes accounted for 76% of the risk of myocardial infarction [5]. Another study that compiled data from 14 clinical trials, involving 122,458 patients, similarly concluded that smoking, diabetes, hyperlipidemia, and hypertension are affected by these same main risk factors [6]. In 2012, it was reported that there was a higher prevalence of CRFs in the UAE, as opposed to other developed countries, and the related deaths were above the global average [7].

Although clinical signs of CVD usually present in adulthood, early atherosclerotic changes occur during adolescence. The Framingham Offspring Study showed that risk factor exposure during early adulthood (ages 20–39 years) was associated with coronary heart events after the age of 40 years [8]. The study showed that high blood pressure, and abnormal lipid levels, were associated with an 8 to 30-fold increase in cardiac events.

The most important behavioral risk factors for CVD that are comprehensively reported in the literature, include: tobacco use, physical inactivity, and poor diet [9–12]. Additionally, there are a number of underlying determinants of CVD, such as socioeconomic status (SES) and hereditary factors. Examples of SES indicators, on the individual level, include: education, occupation and marital status. With regards to education, studies have shown that the higher the education level of the individual is, the greater the possibility of adequate life choices, which in turn leads to a reduced prevalence of hypertension, diabetes and obesity [13–15]. With regards to employment, although evidence that having a financial income can, for example, increase health quality by being able to have better access to healthy food options, however the stress and demands of a job can increase CVD risk by 50% [16]. A meta-analysis on marital status, as a social factor affecting CVD risk, concluded that, being unmarried increased the odds of CVD by 42% and CHD by 16% compared to married individuals [17]. Looking at hereditary factors, the World Heart Federation (WHF) states that if a first-degree relative suffered from a heart attack before the age of 55 for men, or 65 for women, the subject is at greater risk of developing the disease [18].

There are limited studies in the UAE that focus primarily on young adults in the context of CVD and associated risk factors. The objective of this study was to investigate the burden of CRFs in a sample of young Emiratis, and to estimate the prevalence of CRFs within social and behavioral determinants.

## Methods

### Study population

The study sample includes participants from the UAE Healthy Future Study (UAEHFS) [19]. The UAEHFS is a population-based prospective cohort study recruiting 20,000 adult Emiratis to explore the risk factors for NCDs in the UAE. Participants are opportunistically recruited at multiple sites including health centers, universities and companies [19]. The study was based on the cross-sectional analysis of baseline data from the UAEHFS cohort, recruited between February 2016 and December 2018. Subjects were Emirati nationals aged 18 to 40 years. All participants were required to provide informed consent. Participants who reported any acute infection at the time of recruitment and pregnant women were excluded from the study. This study was approved by the Abu Dhabi Health Research and Technology Committee (ref. DOH/HQD/2020/516). Additional information on the UAEHFS methodology is published elsewhere [19].

### Data collection protocol for UAEHFS

Participants answered a self-completed questionnaire, underwent physical measurements, and gave a blood sample. The questionnaire collected information on risk factors that pertain to NCD development. The questions explored socio-demographic factors, general health, and early life exposures. The family history of NCDs was also considered to see whether a parent may have had a heart disease, stroke, or a combination of a known CVD risk factor such as high cholesterol, hypertension, diabetes and obesity. Physical activity was also assessed using the WHO’s physical activity tool; the Global Physical Activity Questionnaire (GPAQ) [20]. The tool quantifies the physical activity levels and time spent into metabolic equivalents that can be calculated and categorized into low, moderate and high physical activity levels.

The self-completed questionnaire also addressed tobacco smoking status and types (cigarette, midwakh, or water-pipe *“shisha”).* The subsequent steps of physical assessments included measuring brachial blood pressure and anthropometric measures (BMI, waist and hip circumferences) were performed by trained nurses that followed a standardized protocol. Finally, a sample of random venous blood was collected and used for analyzing blood lipids and HbA1c. Only fasting blood samples were used to analyze plasma glucose.

### Cardiometabolic risk factors (CRFs)

Body mass index (BMI) was categorized according to the WHO definitions; a BMI less than 25.0 kg/m^2^ was considered normal, a BMI that lies between 25.0 and 29.9 kg/m^2^ was considered overweight, and a BMI above 30.0 Kg/m^2^ was classified as obese. Dysglycemia, or abnormal glycemic status, was defined as having one or more of the following; HbA1c ≥ 5.7%, self-reporting diabetes or taking antidiabetic medication in the questionnaire, or fasting blood glucose (FBG) ≥ 100 mg/dL (this was only done for a subset of 1080 participants).

Dyslipidemia was defined as either self-reported diagnosis of abnormal cholesterol level, or taking a lipid-controlling medication in the questionnaire, or having an abnormal test level of any of the following; low-density lipoprotein (LDL) cholesterol level of ≥130 mg/dL, high-density lipoprotein (HDL) cholesterol level of ≤40 mg/dL for men or ≤ 50 mg/dL for women, total cholesterol ≥200 mg/dL, or triglycerides ≥150 mg/dL for fasting samples and ≥ 175 mg/dL for non-fasting samples [21, 22].

Elevated blood pressure, or hypertension, was defined as having 2 or 3 blood pressure readings of ≥140 mmHg systolic and/or ≥ 90 mmHg diastolic according to the American Heart Association guidelines [23]. Hypertension was also defined as having self-reported “hypertensive” on the questionnaire and/or whether they are taking blood pressure-controlling medication. Central obesity was indicated if the waist-to-hip ratio was ≥0.85 for women and ≥ 0.90 for men [24].

### Statistical analyses

Categorical data was presented as frequencies and percentages. Continuous variables were presented as means ± standard deviation. The frequencies and percentages were tested for significance of any differences in distribution between two or more groups using Chi-square and Fisher’s exact tests, as appropriate. For continuous variables, differences in means were measured by t-tests and one-way ANOVA tests.

Logistic regression models were used to estimate the overall age-adjusted prevalence for each CRF, and to estimate age-adjusted prevalence of every CRF within each social and behavioral factor. Estimates were reported with the 95% confidence interval (95% CI). Two-sided tests with *P* < 0.05 were considered statistically significant. The analyses were performed using Stata statistical software version 15 [25].

## Results

Overall, a total of 5167 subjects aged between 18 and 40 years were recruited between February 2016 and December 2018. Participants were from different cities; with the majority (around 70%) from Abu Dhabi emirate. Complete self-reported data was available for up to 85% of the participants, depending on the data point concerned. Complete body measurements including anthropometrics and blood pressure was available for 94% of the sample. Finally, complete blood sample testing was available for 98% of the sample; where 79.1% were non-fasting and 20.9% were fasting samples.

Table 1 summarizes the study sample’s social and behavioral characteristics. It included 38% females and 62% males. The mean age for the sample was 25.7 (±6.2) years with a median age of 24 years. The age distribution was significantly different between women and men, where women were generally younger in the sample. Most of the participants were single (63.6%) and employed (53.9%). About half of the participants had college or post-graduate degree (46%) while the other half had a high-school diploma or below (54%). Among females, the majority were students (44.3%), while most men were employed (68.5%) (*P* < 0.001). Family history of NCDs was reported by 56% of the overall study population.

**Table 1:** Social and behavioral characteristics of the UAE Healthy Future Study participants

	OVERALL	MEN	WOMEN	***P***-value
	***N*** = 5167	3202 (62%)	1965 (38%)	
**Social factors**				
Age (years), mean (SD)	25.7 (6.2)	26.4 (5.9)	24.5 (6.3)	< 0.001
**Age groups**				
18–19	872 (16.9%)	374 (11.7%)	498 (25.3%)	
20–24	1824 (35.3%)	1091 (34.1%)	733 (37.3%)	
25–29	1068 (20.7%)	778 (24.3%)	290 (14.8%)	
30–34	784 (15.2%)	563 (17.6%)	221 (11.3%)	
35–40	619 (12.0%)	396 (12.4%)	223 (11.4%)	
*Missing* ^a^	*0*	*0*	*0*	
**Marital status**				< 0.001
Single	2804 (63.6%)	1522 (56.2%)	1282 (75.4%)	
Married	1497 (34%)	1144 (42.2%)	353 (20.8%)	
Divorced/widowed	108 (2.5%)	43 (1.6%)	65 (3.8%)	
*Missing* ^a^	*493 (15.4%)*	*265 (13.5%)*	*758 (14.7%)*	
**Employment**				< 0.001
Employed	1978 (53.9%)	1535 (68.5%)	443 (31%)	
Students	1032 (28.1%)	399 (17.8%)	633 (44.3%)	
Unemployed	658 (17.9%)	306 (13.7%)	352 (24.7%)	
*Missing* ^a^	*962 (30.0%)*	*537 (27.3%)*	*1499 (29.0%)*	
**Education level**				< 0.001
High school& below	2326 (54.0%)	1468 (55.3%)	858 (51.9%)	
College & above	1984 (46.0%)	1189 (44.7%)	795 (48.1)	
*Missing*	*545 (17.0%)*	*312 (15.9%)*	*857 (16.6%)*	
**Family history of NCD**				< 0.001
No	2228 (44.0%)	1459 (46.5%)	769 (40.0%)	
Yes	2830 (56.0%)	1677 (53.5%)	1153 (60.0%)	
*Missing* ^a^	*66 (2.1%)*	*43 (2.2%)*	*1.9 (2.1%)*	
**Behavioral factors**				
**Smoking**				
Non-smoker:	2628 (66.9%)	1170 (49.0%)	1458 (94.8%)	< 0.001
Current smoker:	1299 (33.1%)	1219 (51%)	80 (5.2%)	
Cigarette	666 (17.7%)	643 (28.5%)	23 (1.5%)	
Midwakh	802 (21.2%)	779 (34.3%)	23 (1.5%)	
Shisha	791 (21.2%)	722 (32.3%)	69 (4.6%)	
*Missing* ^a^	*813 (25.4%)*	*427 (21.7%)*	*1240 (24.0%)*	
**Physical activity**				
**Metabolic Equivalent** (minutes/week), mean (SD)	5514.3 (6306.8)	6456.4 (7003.2)	4129.9 (4792.4)	< 0.001
Low	1894 (80.9%)	1119 (80.3%)	775 (81.8%)	< 0.001
Moderate	189 (8.1%)	95 (6.8%)	94 (9.9%)	
High	258 (11%)	179 (12.9%)	79 (8.3%)	
*Missing* ^a^	*1809 (56.5%)*	*1017 (51.8%)*	*2826 (54.7%)*	

Data is presented as mean values with standard deviation (SD) or frequency numbers (N) and percentages (%). *P*-values are derived from t-tests for continuous measures and chi-square tests for categorical variables

^a^Missing numbers were not included in the column percentages

Smoking was self-reported in 33.1% of the study sample, including three different types of tobacco smoking; cigarette, shisha, and midwakh. There were more male smokers (51%) than female smokers (5.2%) (*P* < 0.001). In men, the prevalence of tobacco use was similar amongst the three types of tobacco smoking. However, smoking shisha was more common than smoking other types of tobacco amongst women. For physical activity, approximately 81% were categorized as performing low-physical activity and 19% as moderate-to-high physical activity. Men reported higher number of active minutes per week compared to females (*P* < 0.001) (Table 1).

The mean values of cardiometabolic markers of the study sample are presented in Table 2. All biomarkers were significantly higher in men than women (*P* < 0.001) with an inverse in high-density lipoprotein (HDL). In the overall sample, obesity was estimated as 27.2% (25.9–28.4), the age-adjusted prevalence was 26.5% (25.2–27.7). Based on HbA1c analysis, 6.5% of the sample had prediabetes and 1.9% had diabetes. Fasting serum glucose yielded a prevalence of 17.8% for prediabetes and 2.7% for diabetes. Together, glycated hemoglobin, fasting blood glucose and self-reported diagnosis or medication identified prediabetes prevalence as 8.2% and diabetes as 3.5%. The overall prevalence of dysglycemia (prediabetes and diabetes) was 12.5% (11.7–13.25); and the age-adjusted prevalence was 11.7% (10.8–12.7), as presented in Table 3.

**Table 2:** Mean values of cardiometabolic markers of the UAE Healthy Future Study participants

Cardiometabolic markers	Overall, Mean (SD)	Men, Mean (SD)	Women, Mean (SD)	***P***-value
Body Mass Index (BMI), kg/m^2^	26.9 (6.3)	27.7 (6.0)	25.8 (6.6)	< 0.001
Waist – hip ratio	0.82 (0.09)	0.87 (0.07)	0.77 (0.08)	< 0.001
Systolic blood pressure, mmHg	126.0 (14.1)	131.2 (13.1)	117.8 (11.6)	< 0.001
Diastolic blood pressure, mmHg	78.0 (77.7)	80.3 (10.2)	74.3 (8.6)	< 0.001
Low-density lipoprotein (LDL), mg/dL	115.9 (34.0)	122.0 (35.8)	105.9 (28.1)	< 0.001
High-density lipoprotein (HDL), mg/dL	48.4 (12.9)	43.9 (10.6)	55.7 (13.0)	< 0.001
Total cholesterol, mg/dL	182.5 (36.0)	185.8 (38.6)	177.0 (30.3)	< 0.001
Triglycerides (TG), mg/dL	103.9 (77.6)	118.8 (86.0)	79.3 (52.8)	< 0.001
Glycated hemoglobin (HbA1c), %	5.26 (0.66)	5.29 (0.71)	5.21 (0.58)	< 0.001
Fasting blood glucose (FBG), mg/dL	94.3 (25)	96.4 (25.7)	88.8 (22.3)	< 0.001

Data is presented as means (standard deviation). *P*-values are derived from t-tests

**Table 3:** Age-adjusted prevalence of cardiometabolic risk factors of the UAE Healthy Future Study participants

CRFs	Overall	Men	Women	***P*** value
**Obesity**	26.5 (25.2–27.7)	29.7 (28–31.4)	21.6 (19.7–23.5)	< 0.001
**Dysglycemia**	11.7 (10.8–12.7)	14.0 (12.7–15.2)	8.3 (7.0–9.6)	< 0.001
**Dyslipidemia**	62.7 (61.3–64.0)	68.0 (66.3–69.7)	54.2 (52.0–56.5)	< 0.001
**Hypertension**	22.4 (21.2–23.6)	30.9 (29.2–32.6)	9.2 (7.8–10.5)	< 0.001
**Central obesity**	22.5 (21.3–23.8)	29.6 (27.9–31.3)	12.5 (10.9–14.0)	< 0.001

Data is presented as percentage (95% CI)

Dyslipidemia was reported in 62.7% (61.3–64.0) of the study sample, and it was higher in men (68.0% (66.3–69.7)) than women ((54.2% (52.0–56.5)) (*P* < 0.001). The mean systolic and diastolic blood pressures were significantly different between men and women (*P* < 0.001). The overall age-adjusted prevalence of hypertension was observed in 22.4% (21.2–23.6) of the sample; 30.9% (29.2–32.6) in men and 9.2% (7.8–10.5) in women. Finally, age-adjusted central obesity was 22.5% (21.3–23.8). Nearly a third of men had an increased waist-to-hip ratio (29.6% (27.9–31.3)), while only 12.5% (10.9–14.0) of women had central obesity.

Table 3 summarizes the age-adjusted prevalence for the CRFs. The prevalence for each of the five CRFs are significantly different across age groups in men and women, as visualized in Supplementary Fig. 1.

The age-adjusted distribution of the CRFs was assessed within the social and behavioral determinants in men and women; presented in Tables 4 and 5. In men, smokers had higher prevalence of obesity, dyslipidemia, and central obesity than non-smokers (*p* < 0.05). Men in the lower education group had higher obesity and hypertension cases than men within higher education groups. Whereas, unemployed men had higher dysglycemia than students. Additionally, single or divorced men tended to be more hypertensive than married men.

**Table 4:** Age-adjusted prevalence of CRFs by social and behavioral determinants in men

MEN					
	Obesity	Dysglycemia	Dyslipidemia	Hypertension	Central obesity
**Marital Status**					
Single/divorced	29.1 (26.4–31.8)	14.1 (12.1–16.1)	68.3 (65.5–71.2)	33.8 (31–36.6)^a^	27.8 (25.1–30.5)
Married	29.7 (26.4–32.9)	15.8 (13.2–18.3)	67.5 (64–70.9)	28.6 (25.4–31.8)^a^	30.8 (27.4–34.1)
**Employment Status**					
Unemployed	29.1 (23.5–34.6)	17.8 (13.1–17.7)^a^	68.2 (62.8–73.6)	30.8 (25.5–36.1)	27.0 (22.0–32.1)
Employed	29.8 (27.3–32.4)	15.7 (13.7–17.7)	68.2 (65.7–70.7)	33.6 (31.1–36.0)	36 (33.6–38.5)
Student	31.8 (26.3–37.3)	9.4 (5.9–12.8)^a^	69.8 (64.8–74.8)	26.2 (21.8–30.5)	18.6 (14.7–22.4)
**Education level**					
High School & below	32.7 (30.2–35.2)^a^	15.7 (13.8–17.6)	68.6 (66–71.2)	33.7 (31.2–36.3)^a^	30.6 (28–33.1)
College & above	25.1 (22.6–27.7)^a^	13.7 (11.7–15.6)	67.1 (64.3–70)	28.6 (25.9–31.2)^a^	27.8 (25.1–30.5)
**Family history of NCDs**					
No	27.1 (24.7–29.6)^a^	12.1 (10.5–13.8)^a^	65.7 (63.2–68.3)^a^	25.7 (23.3–28.1)^a^	29.3 (26.7–31.9)
Yes	31.8 (29.5–34.1)^a^	15.6 (13.9–17.4)^a^	69.7 (67.4–72)^a^	35.1 (32.8–37.4)^a^	29.8 (27.5–32.1)
**Smoking**					
Non-smoking	27.4 (24.7–30)^a^	15.1 (13–17.2)	63.6 (60.7–66.5)^a^	32.7 (30–35.5)	26.4 (23.7–29.1)^a^
Smoking	31.2 (28.5–33.9)^a^	14.4 (12.4–16.4)	71.1 (68.5–73.8)^a^	31.0 (28.3–33.6)	31.9 (29.2–34.7)^a^
**Physical Activity**					
Moderate/High	25.2 (20–30.5)	11 (7.3–14.6)	66.8 (61–72.6)	33.1 (27.5–38.8)	22.9 (17.7–28)
Low PA	27.9 (25.2–30.6)	12 (10.1–14)	66.3 (63.4–69.2)	30.8 (28.1–33.6)	28.3 (25.5–31.1)

Data is presented as percentages (95% CI). Percentages are derived from logistic regression analyses adjusting for age

^a^have significant difference in proportions of CRFs (*P* < 0.05)

**Table 5:** Age-adjusted prevalence of cardiometabolic risk factors by social and behavioral determinants in women

WOMEN					
	Obesity	Dysglycemia	Dyslipidemia	Hypertension	Central obesity
**Marital Status**					
Single/divorced	20.7 (18.5–23.0)	7.6 (6.1–9.0)	53.4 (50.6–56.2)	9.8 (8.1–11.4)	12.1 (10.2–13.9)
Married	22.3 (17.5–27.0)	10.4 (7.1–13.8)	60.6 (54.6–66.6)	9.0 (5.9–12.1)	12.6 (9.0–16.1)
**Employment Status**					
Unemployed	24.5 (19.8–29.1)^a^	11.3 (8–14.7)^a^	61.6 (56.4–66.8)	11.5 (8.1–15.0)	14.2 (10.5–17.9)
Employed	23.9 (19.2–28.5)	10.1 (6.9–13.3)	51.9 (46.3–57.6)	9.8 (6.6–12.9)	13.2 (9.6–16.8)
Student	17.7 (14.0–21.3)^a^	4.8 (2.8–6.7)^a^	53.6 (48.9–58.3)	10.0 (7.0–12.9)	10.6 (7.5–13.6)
**Education level**					
High school & below	25.9 (22.7–29.0)^a^	9.4 (7.4–11.5)^a^	59.0 (55.6–62.4)^a^	10.5 (8.4–12.7)	14.3 (11.8–16.8)^a^
College & above	15.8 (13.1–18.4)^a^	6.7 (4.9–8.4)^a^	50.0 (46.4–53.7)^a^	8.8 (6.8–10.9)	10.2 (8.1–12.3)^a^
**Family history of NCDs**					
No	18.2 (15.34–21.0)^a^	6.6 (4.8–8.3)^a^	50.3 (46.7–53.9)^a^	7.0 (5.1–8.8)^a^	11.3 (9.0–13.7)
Yes	24.2 (21.6–26.7)^a^	9.4 (7.7–11.1)^a^	57.2 (54.3–60.1)^a^	10.9 (9.0–12.8)^a^	13.2 (11.1–15.2)
**Smoking**					
Non-smoking	20.2 (18.1–22.4)	7.6 (6.2–9.0)	54.2 (51.6–56.8)	9.5 (7.9–11.1)	11.3 (9.6–13)
Smoking	28.9 (18.9–38.9)	10.7 (4.3–17.2)	57.3 (46.3–68.3)	14.0 (6.5–21.5)	13.9 (6.6–21.2)
**Physical Activity**					
Moderate/High	21.9 (15.5–28.3)	9.0 (4.7–13.4)	58.4 (50.9–65.8)	10.7 (5.9–15.5)	7.7 (3.7–11.8)
Low PA	19.3 (16.5–22.2)	6.5 (4.7–8.3)	52.4 (48.8–55.9)	9.5 (7.4–11.7)	10.2 (7.9–12.4)

Data is presented as percentages (95% CI). Percentages are derived from logistic regression analyses adjusting for age

^a^have significant difference in proportions of CRFs (*P* < 0.05)

In women, obesity and dysglycemia were significantly higher in the unemployed group compared to students (*p* < 0.05). Women in the lower education group reported significantly higher prevalence of all CRFs, except for hypertension, than those in the higher education one (*p* < 0.05). Interestingly, obesity, dysglycemia, dyslipidemia and hypertension were higher in the more the physically active group, however these findings were not statistically significant. All CRFs, with the exception of central obesity, across both sexes were significantly higher among participants with family history of NCDs (*P* < 0.05).

## Discussion

This study presents the first comprehensive epidemiological assessment of the major CRFs in a large sample of young Emirati adults, including obesity, dysglycemia, dyslipidemia, hypertension and central obesity. All CRFs were highly prevalent across the whole sample, but significantly higher in men compared to women. This study investigated, for the first time, how CRFs prevalence differs among different social and behavioral determinants.

Obesity was present in 26.5% of our population. This estimate was similar to the prevalence reported in earlier work, where the prevalence estimates of obesity ranged from 25 to 35.4% in similar age groups [4, 26–28]. In this study, obesity was higher in men than in women. This trend was similar to another nation-wide study published in 2012 [26]. A review led by Azizi et al. on the metabolic health status in the Middle East and North Africa (MENA) region projected a further increase in high BMI prevalence in 2025 to 36.3% (25.0–48.5) in men and 47.8% (37.1–58.9) in women [29].

The age-adjusted prevalence for pre-diabetes was 8.2% and for diabetes was 3.5% in the whole study population. These prevalence estimates were similar to the estimates reported by the UAE national survey for the age group 18–44 years; where diabetes had a prevalence of 3.3% and prediabetes was 6.5% among Emiratis [4]. The study findings showed that the age-adjusted prevalence of dysglycemia in this population was 11.7% and it was higher in males (14.0%) than in females (8.3%). In this analysis, the prevalence of dysglycemia doubled from the youngest age group (below 20 years) to the oldest age group (35 to 40 years) (*p* < 0.01). It was found that 7.6% of participants aged 18 and 19 years, and 8.3% of participants between 20 and 24 years had abnormal glycemic status. This supports the international connotation that prediabetes and diabetes are rapidly rising in the adolescents and young adults as reported by the Centre for Disease Control and Prevention (CDC) [30]. According to the International Diabetes Federation (IDF), the age-adjusted prevalence of diabetes was 16.3% in UAE, while it is 12.2% in the Middle East and North Africa (MENA) region in 2019 [31]. The MENA region had the highest prevalence compared to other parts of the world. A recent analysis on 33,000 men in the UAE revealed a relatively higher prediabetes prevalence of 33% in the 18–19-year-old age group, and 40.2% in the 20–24-year-old age group based on fasting blood glucose measurements [27]. Projected estimates of 2025 state that diabetes will increase to 19.9% (8.0–41.1) in men and women [29].

With the broad definition of dyslipidemia applied in this study, the results revealed that 62.7% of the whole sample had abnormal lipid profiles. This high proportion of dyslipidemia might not be comparable to other local studies due to the difference in the definition criteria and methods of blood sampling; fasting or random [26, 27]. The global prevalence of dyslipidemia among adults was reported as high as 39% in 2008 [32]. They showed that the prevalence of dyslipidemia was positively associated with the income of the country and estimates were double in high-income countries compared to low-income countries.

Elevated blood pressure was identified in 22.4% of the sample. Hypertension in men was 3-folds higher than in women, 30.9% versus 9.2%, respectively. In men, hypertension was highest (30.0%) in the 20–24 age group, whereas in women, the prevalence was highest (25%) in the oldest age group; 35–40 years. In line with other reports, men consistently had a higher prevalence for hypertension than women [4, 26]. A global prevalence of 26.4% was estimated among adults in year 2000 [33]. In the age and gender breakdown, hypertension was reported in 12.7% among the 20–29-year-old age group and 18.4% in the 30–39-year-old age group in men. Men had double the rates reported for women in all age groups. NCD trends in UAE show that hypertension prevalence decreases when compared to data from 1975 to 2015 [29]. A possible explanation to the reduction, despite unfavorable trends in sodium intake, obesity and physical inactivity, maybe be due to the use of antihypertensive drugs among other unknown factors.

The prevalence of abdominal obesity in this study population was estimated as 24.3%, and males had a higher prevalence than women; 29.6 and 12.5%, respectively. However, these findings were lower than that of the Weqaya study, where the prevalence for abdominal obesity was 46.5% in men and 36.4% in women, aged 18–39 years [26]. In a smaller local study on young women aged 18–25 years, high waist circumference was detected in 18.2% of the sample [34]. In the US, the National Health and Nutrition Examination Survey (NHANES) report of 2007–2010 estimated abdominal obesity in 18–39 year old age group as 38.7% [35].

Global patterns of abdominal obesity show that women generally have higher prevalence than men [36]. The Weqaya study showed that, by stratifying by age and gender, women in the younger age-groups had lower rates of central obesity than men. However, in the sixty age-group, women shifted to have higher rates of central obesity than men [26]. This could be explained by the effect of menopause. The literature shows that central obesity is also associated with low levels of testosterone; a hormone that promotes fat metabolism and decreases central obesity [37, 38]. However, this pattern was not detected in our young sample below the age of 40 years.

To exclude collinearity between obesity and central obesity, correlation tests between BMI and waist-to-hip ratio were carried out; the estimated correlation was 0.42. We found that among individuals that did not have central obesity, 18.5% were BMI-obese. As for those that did have central obesity, only 54% had BMI-obesity.

In this study, we also investigated the CRFs distribution within different social characteristics, such as marital status, employment and educational attainment, as well as behavioral determinants, such as smoking and physical activity. A positive family history of NCDs was also investigated. In men, the prevalence of obesity, dyslipidemia and central obesity were higher among smokers. Although obesity is usually lower among smokers than non-smokers, this was not the case in our sample. Obesity was significantly higher in smokers 31.2% (28.5–33.9) versus 27.4% (24.7–30.0) in non-smokers. This finding is in accordance with Sulaiman et al.’s report where smokers had higher BMI than non-smokers; 28.7% vs. 20.7% respectively [39]. Similarly, the Northern Finland Birth Cohort 1966 study sample showed that smokers had a higher BMI, waist circumference, dyslipidemia and hypertension when compared to non-smokers [40].

Furthermore, lower education attainment in both men and women showed significantly higher CRFs; 2 out of 5 the CRFs in men and 4 out of the 5 CRFs in women. Similarly, in a study that investigated the relationship between education and CVD incidence, those with higher education (of a university degree) had a smaller percentage of people with hypertension, BMI and diabetes compared to people with lower education (*P* < 0.001) [41]. Unemployed men and women had a higher prevalence of dysglycemia than in students. This finding can be supported by Rautio et al.’s [42] conclusion that unemployment was related to prediabetes and diabetes.

Both men and women with a positive family history of NCDs had significantly higher prevalence of CRFs compared to those with no family history (*P* < 0.05). It is well established that family history of disease and metabolic abnormality play a big role on offspring, due to the combination of both genetic and environmental factors [43]. There was no significant difference in the distribution of risk factors according to physical activity levels. This could be attributable to the fact that 81% of the sample were classified as low physically active.

This study used a broad definition for dyslipidemia, which was based on four lipid markers, self-report and the use of lipid-lowering medication. This definition was recommended by the ATP3 guidelines for persons above 20 years old [44]. We also used random non-fasting samples for the analysis, which is unconventional to normal practice. Traditionally, blood collection for lipid testing purposes is required to be fasting samples. However, recent reports show that random blood samples are acceptable. Observational studies demonstrate that in comparison to fasting level, measurements only altered minimally, by 8 mg/dL or 0.2 mmol/L, when compared to fasting lipid levels [45]. So far, there is no robust -scientific evidence to why fasting samples are better than random samples when evaluating lipid profile for cardiovascular risk prediction. In fact, most studies now recommend non-fasting samples as they are easier to collect during the day and represent the normal postprandial state of individuals. Many countries are now changing their guidelines towards a consensus on measuring lipid profiles for cardiovascular risk prediction in the non-fasting state to simplify blood sampling for patients, laboratories, and clinicians worldwide [46].

The principal strengths of this study include the large sample size of young Emiratis, and the extensive information collected. This study mainly focused on recruiting young adults, who are often underrepresented in other non-communicable disease studies. Another strength is the thorough process, the use of objective tools and the various data points, from sociodemographic, to lifestyle behaviors, health and family history. All blood samples and physical measurements were collected in a standardized procedure to ensure consistent quality and reduce the risk of information bias. All of these data points allowed us to employ detailed and specific disease-identification criteria.

The main weakness of this study is that it is based on opportunistic recruitment of study volunteers. This might introduce the risk of having selection bias and potentially affect the representativeness of the study sample. Another limitation observed is that more males (62%) were recruited than females (38%), and that they were recruited from different centers. The analysis of the results therefore varied and were described separately for each gender. Moreover, it is essential to address a major limitation related to cross-sectional studies, which is the inability to identify a causal relationship between the potential risk factor and outcome. Therefore, the results of this study must be interpreted cautiously and inferred to the local populations of similar age and sociodemographic characteristics. Similar to other observational studies, this study is prone to measurement and recall bias.

Moreover, the high number of missing data for physical activity have possibly affected the capability to capture a relationship to CRFs in the model. Although validation studies conclude that generally GPAQ is an acceptable measure of physical activity; results ranged between fair-to-moderate validity [47, 48]. However, it does not adequately assess sedentary behavior. Sedentary behavior is not synonymous with physical inactivity. An individual can be physically active, and have long hours of sedentary behavior [49]. Therefore, it is important to address sedentary behavior independently from physical activity. Finally, the lack of dietary data, which is another important behavioral risk factor that is known to affect cardiometabolic health, was another limiting factor to the study.

## Conclusion

This study on cardiometabolic risk factors provided thorough information about the cardiovascular risk in young adults of the United Arab Emirates, which represent the majority age demographic of the country, where 95% of the UAE population is younger than 40 years. This study suggests that the prevalence of obesity, dysglycemia, dyslipidemia, hypertension and central obesity are high. The study showed variation in the distribution of CRFs by social and behavioral characteristics. Understanding that some social groups are more prone for developing a metabolic abnormality can help design specific prevention measures towards them.

## Supplementary Information

**Additional file 1: Supplementary Fig. 1.** Cardiometabolic risk factors across age groups of the UAE Healthy Future Study Participants.

## Data Availability

Data is available upon request.

## Abbreviations

BMI: Body Mass Index
CHD: Coronary heart disease
Chol: Cholesterol
CRFs: Cardiometabolic risk factors
CVD: Cardiovascular disease
FBG: Fasting blood glucose
GPAQ: Global Physical Activity Questionnaire
HbA1c: Hemoglobin A1C
HDL: High density lipoprotein
IDF: International Diabetes Federation
LDL: Low density lipoprotein
METs: Metabolic equivalents
NCD: Noncommunicable disease
SES: Socioeconomic status
TG: Triglycerides
UAE: United Arab Emirates
UAEHFS: UAE Healthy Future Study
WHO: World Health Organization
