# Supplementary material for: High prevalence of cardiometabolic risk factors amongst young adults in the United Arab Emirates: the UAE Healthy Future Study
Source: BMC Cardiovasc Disord. 2023 Mar 15;23:137. doi: 10.1186/s12872-023-03165-3 (PMC10015775; doi:10.1186/s12872-023-03165-3)
Supplement: Supplementary file 1 — Additional file 1: Supplementary Fig. 1. Cardiometabolic risk factors across age groups of the UAE Healthy Future Study Participants. [file 12872_2023_3165_MOESM1_ESM.docx]

*P*<0.001


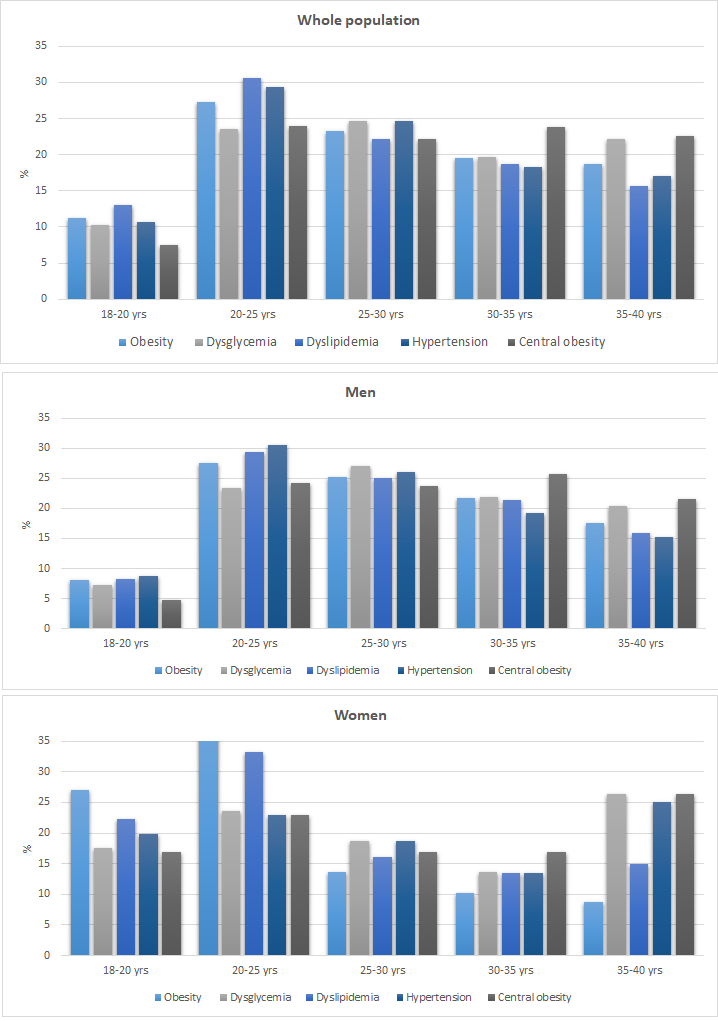


Supplementary Figure 1: Cardiometabolic risk factors across age groups of the UAE Healthy Future Study Participants.

P value 0.00
